# Re-entrant spin glass transitions: new insights from acoustic absorption by domain walls

**DOI:** 10.1038/s41598-017-17297-y

**Published:** 2017-12-04

**Authors:** S. Kustov, J. Torrens-Serra, E. K. H. Salje, D. N. Beshers

**Affiliations:** 1 Universitat de les Illes Balears, Dep.de Física, Cra. Valldemossa, km. 7.5, 07122 Palma de Mallorca, Spain; 2 ITMO University, 49 Kronverkskiy av., St. Petersburg, 197101 Russia; 3 Cambridge University, Department of Earth Sciences, Cambridge, CB2 3EQ United Kingdom; 4 Columbia University, Dept. of Applied Physics and Applied Mathematics, New York, NY 10027 USA

## Abstract

Re-entrant spin glass (RSG) transitions in Ni-Mn and Au-Fe have been reassessed by acoustic measurements of the magneto-mechanical damping by domain walls. Stress-induced non-thermally activated domain wall dynamics is progressively replaced by an intense thermally activated relaxational response when the temperature approaches the RSG freezing point. A “frozen” state with negligible motion of domain walls on atomic and mesoscopic scales occurs in the RSG. We propose that RSG freezing has its origin in intrinsic properties of domain walls.

## Introduction

Re-entrant spin glasses, RSGs, unlike ordinary spin glasses, SG^1–3^, are ferromagnetic at all temperatures below their Curie temperature, *T*_*C*_, but they transform at a still lower temperature, *T*_*f*_, to a frozen state. The real part of the susceptibility, *χ*′(*T*), which shows a cusp during the SG transition is replaced at *T*_*f*_ by a decay of the ferromagnetic moment^3^ while the maximum in the imaginary part, *χ*″(*T*), is maintained. The very nature of the RSG transition is controversial. One model claims that RSG is simply a mixture of ferromagnetic and SG states with spin glass-like clusters embedded in a network of ferromagnetic domains^1–12^. Freezing of the clusters may generate random fields, provoking the breakdown of the ferromagnetic long-range order^13^. These magnetic clusters have not been observed experimentally, however. The co-existence of ferromagnetism and glass-like states may relate to atomic scale chemical disorder.

Another line of thinking, not invoking atomic-scale structural disorder, considers the role of magnetic domain walls in RSG materials, which were observed above and below *T*_*f*_^14,15^. This model involves a strong decrease of the domain wall mobility^15^ and explains the near vanishing of *χ*′. Similarly, the RSG-like behavior of high quality stoichiometric single crystals of magnetite has been explained by pinning of domain walls by unknown centers able to migrate and pin domain walls at temperatures around 25 K^16^. The RSG freezing around 40–50 K in Fe-Ni invar alloys^17–20^ has also been attributed to pinning by static defects such as grain boundaries or compositional fluctuations at the scale of the domain wall width. The wide scope of possible candidates for “pinners” during RSG freezing exemplifies the difficulties in identifying their possible origin. In addition to uncertainties about the pinning mechanism, the domain wall pinning concept does not explain the existence of “frustrated spins” in disordered RSGs. Finally, a similar line of thought, recently introduced in analogy with ferroelectrics^21,22^, involves a generic concept of “domain glasses”. It proposes that glassy behaviour does not necessarily require atomic-scale disorder, but can originate from extended defects in ferroic microstructure, such as domain walls and their mutual interaction, and internal substructures such as Bloch lines and Bloch points. We will argue in this paper that intrinsic freezing of domain wall is the origin of RSG transition.

To clarify the involvement of domains walls in RSGs, we conducted new acoustic experiments studying the magneto-mechanical damping (MMD), which will be expressed as logarithmic decrement of oscillations *δ*. Damping (the imaginary part of the modulus) is particularly sensitive to the DW dynamics: the ideal material has a finite modulus and no losses. Thus a small change in the dynamics will yield only a small change in the modulus relative to the ideal modulus while a small change in the imaginary part of the modulus may be large compared to zero. MMD is defined as any damping that is suppressed by an external magnetic field. In the present context, linear damping *δ*_*i*_ is defined as being independent of the vibrational strain amplitude *ε*_0_. The components of MMD are^23–26^:

- a linear micro eddy current damping, *δ*_*μ*_, measured at low strain amplitudes,
- a linear macro eddy current damping component, *δ*_*M*_, and
- a non-linear hysteretic damping, *δ*_*h*_(*ε*_0_), emerging at higher strain amplitudes.

These components are associated with different length scales: *δ*_*μ*_ is commonly ascribed to short-range reversible domain wall displacements (much) less than the average dimensions of magnetic domains, *δ*_*h*_ is associated with larger-scale irreversible domain wall motions, more comparable with domain sizes. *δ*_*M*_ is measured at a scale comparable with the penetration depth of the macroscopic electromagnetic wave, averaging over many domains. All three MMD components depend on *H* and vanish at saturation. *δ*_*i*_ and *δ*_*h*_(*ε*_0_) display their maximum values either for zero magnetization *M* = 0, *H* = 0, or in its vicinity, then decline monotonously with applied field *H* ^26–28^. *δ*_*M*_ has its maximum value at *H* roughly equal half the saturating field; for *M* = 0, *δ*_*M*_ = 0^27,29^. Thus, detailed studies of MMD components versus temperature, field and strain amplitude show aspects of the domain wall dynamics during RSG freezing at various length scales. Furthermore, *δ*_*μ*_ shows a Debye type maximum at frequencies *f* at ca.10^5^–10^6^ Hz^30^, and is usually negligibly small below 10^3^ Hz and above 10^7^ Hz. Here, in contrast to previous acoustic studies of SG and RSG freezing^31–36^, we use the frequency (~10^5^ Hz) best suited for studying intrinsic properties of domain walls through *δ*_*μ*_. We report results for three typical RSGs, namely Ni_80_Mn_20_, Ni_77_Mn_23_ and Au_86_Fe_14_.

## Experimental Results

**Figure 1:**
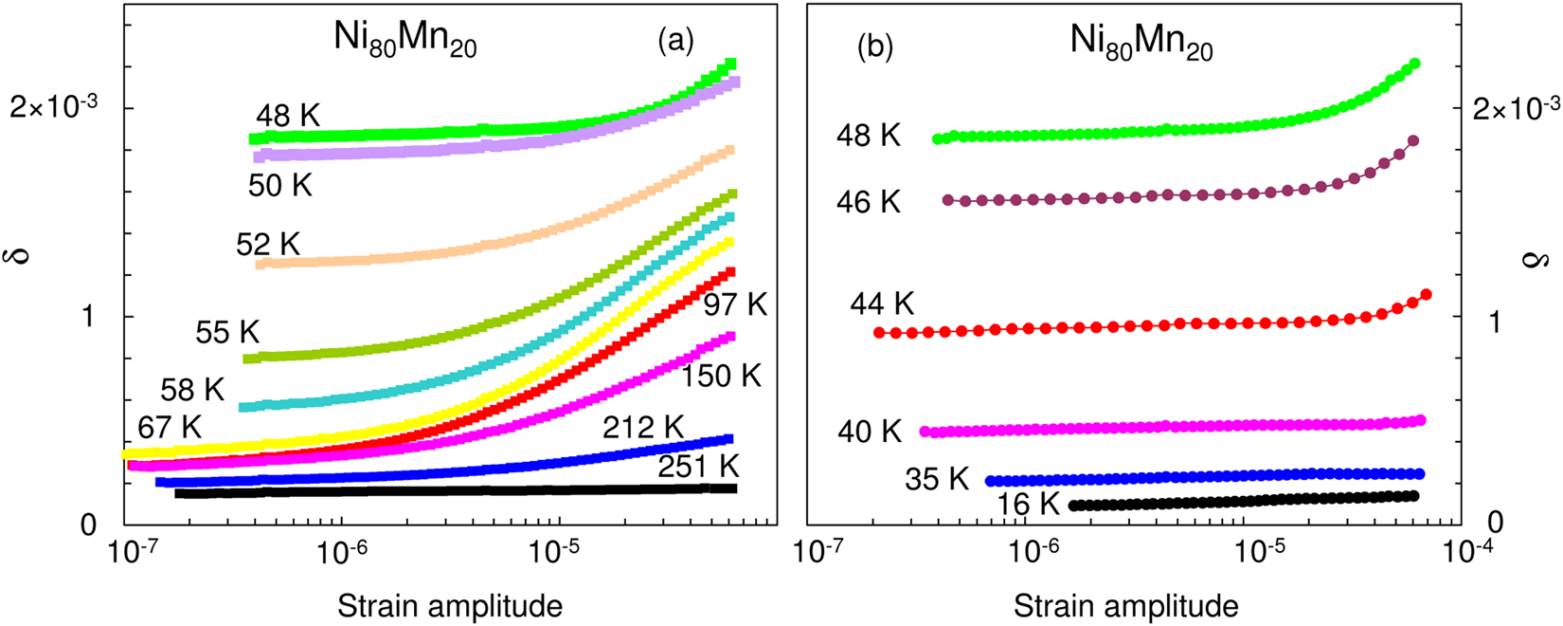
Dependence of the logarithmic decrement in Ni_80_Mn_20_, *δ*, on the strain amplitude at temperatures between 251 K and 48 K (**a**), and between 48 K and 16 K (**b**). Measurements during heating, and at increasing oscillatory strain amplitude with *H* = 0 and magnetization *M* = 0.

Figure 1 shows the variation of *δ* with strain amplitude *ε*_0_ for Ni_80_Mn_20_ measured at 251 K > T > 16 K. The damping remains reasonably linear for strain amplitudes ε_0_ < 10^−6^ over the full temperature range. A strong non-linearity emerges above *ε*_0_ ≈ 10^−6^ between 251 and 212 K. The non-linear damping component is *δ*_*h*_(*ε*_0_) = *δ*(*ε*_0_) − *δ*_*i*_. It reaches a maximum at ca. 70 K (Fig. 1a) and disappears below 40 K, Fig. 1b. A similar trend is shown by *δ*_*i*_ with a maximum close to 48 K.

**Figure 2:**
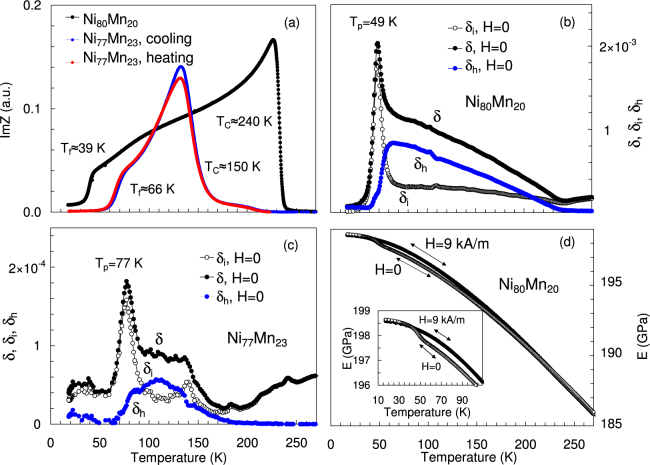
Temperature dependence of elastic, anelastic and electrical properties of Ni_80_ Mn_20_ and Ni_77_Mn_23_ alloys. (**a**) Imaginary part of AC electric impedance, Im(*Z*), for samples of Ni_80_Mn_20_ (heating, frequency 217 Hz) and Ni_77_Mn_23_ (cooling and heating, frequency 686 Hz) alloys. (**b**) The total decrement *δ* of Ni_80_Mn_20_ at strain amplitude of 4 × 10^−5^ and its components, *δ*_*i*_ and *δ*_*h*_ = *δ* − *δ*_*i*_. δ_*i*_ was measured under low strain amplitude of 10^−6^ under heating. (**c**) Same as (**b**) for the sample of Ni_77_Mn_23_ alloy, during cooling. (**d**) Young’s modulus *E* of the sample of Ni_80_Mn_20_ alloy on cooling and heating under zero field *H* = 0 and polarizing field *H* = 9 kA/m, oscillatory strain amplitude *ε*_0_ = 10^−6^. The inset shows details of the temperature spectra around the freezing temperature.

**Figure 3:**
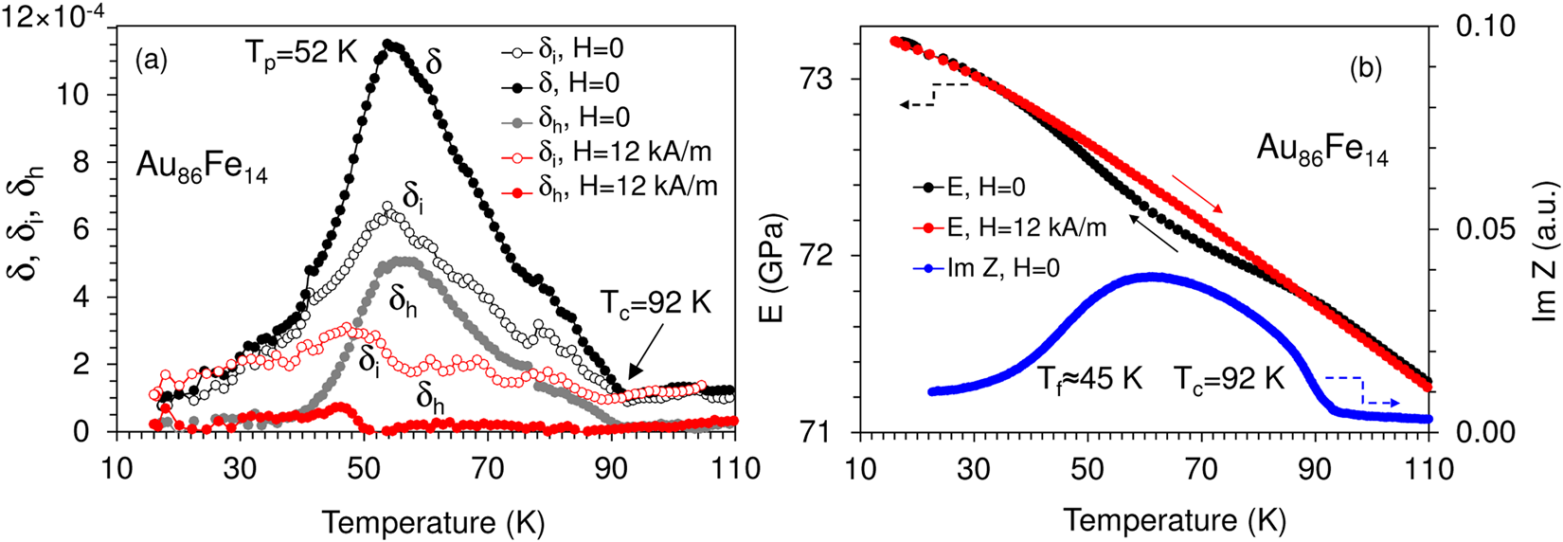
Temperature dependences of some elastic, an-elastic and electrical properties of Au_86_Fe_14_. (**a**) *δ* at a strain amplitude of 2 × 10^−5^, *δ*_*i*_ at a strain amplitude of 10^−6^; *δ*_*h*_ with *H* = 0, during heating; *δ*_*i*_ and *δ*_*h*_ are shown also under *H* = 12 kA/m. (**b**) Im(*Z*) at 686 Hz and *E*(*T*,*H*) during cooling (H = 0), and subsequent heating (*H* = 12 kA/m) for *E*(*T*,*H*).

Figures 2 and 3 display the temperature dependences of elastic, anelastic properties and Im(*Z*) for the two Ni-Mn alloys (Fig. 2) and an Au_86_Fe_14_ sample (Fig. 3).

We determine *T*_*C*_ of the paramagnetic to ferromagnetic transition for all samples using three indicators (Figs 2 and 3): (i) the intercept of the steepest negative slope of Im(*Z*[*T*]) with the temperature axis (Fig. 2a)^37,38^, (ii) the discontinuity in *dδ*_*i*_/*dT* (Figs 2b and 3a), (iii) the emerging of *δ*_*h*_ (Figs 2b and 3a)^38^. All three parameters yield the same *T*_*C*_ for Ni_80_Mn_20_ and for Au_86_Fe_14_ but there is some spread for Ni_77_Mn_23_, possibly due to sample inhomogeneity. The RSG freezing temperatures *T*_*f*_ are determined in two ways: (i) from the maximum slopes of the temperature dependences of Im(*Z*) and *δ*_*h*_ (maximum freezing rate at *T*_*f*_); (ii) the intercepts of the steepest slopes of Im(*Z*) and *δ*_*h*_ versus *T* with the temperature axis. The latter temperatures, *T*_*f*_ *, correspond to a “frozen” state of the system. A comparison of these values, obtained from Im(*Z*) (AC permeability) and damping data are given in Table 1 for the three alloys.

**Table 1:** RSG freezing temperatures determined from the AC impedance data (frequencies 217 and 686 Hz) and from damping data (frequency 91 × 10^3^ Hz).

Alloy	Frequency (Hz)	*T*_*f*_ (K)	*T*_*f*_ *(K)
Ni_80_Mn_20_	217	40	30
Ni_80_Mn_20_	91 × 10^3^	49	43
Ni_77_Mn_23_	686	69	59
Ni_77_Mn_23_	91 × 10^3^	77	65
Au_86_Fe_14_	686	46	31
Au_86_Fe_14_	91 × 10^3^	53	41

The frequency dispersion of RSG freezing is seen by the shift of the ultrasonic freezing temperature at frequency ~10^5^ Hz to lower temperatures at ~10^2^ Hz. The non-magnetic origin of the damping below *T*_*f*_ is seen by the *H* independence of the low-temperature background (Fig. 3a). Figures 2d and 3b show the overall increase of the Young’s modulus *E* with decreasing *T*. E depends on the applied field *H* below *T*_*C*_. The difference between *E* at saturation and in the demagnetized state represents the magnetic contribution to the modulus defect, ${\rm{\Delta }}E/E$, (the *ΔE*-effect^25,29^). The *E*(*T*) dependences for *H* = 0 and large *H* coincide and ${\rm{\Delta }}E/E$ converges to zero near *T*_*f*_. ${\rm{\Delta }}E/E$ measures the in-phase component of the domain wall-related nonelastic strain in mechanical experiments and *δ* - the out-of-phase component. The nonelastic strain vanishes when both ${\rm{\Delta }}E/E$ and *δ* vanish (as in Ni_80_Mn_20_, Fig. 2d and Au_86_Fe_14_, Fig. 3b) showing freezing of magnetomechanical anelastic effects below *T*_*f*_. The frequency shift of the freezing *δ*_*i*_ peak (Table 1) and the concomitant Young’s modulus variation (Figs 2d and 3b) identify this peak as a thermally activated relaxation. This relaxational damping is denoted by *δ*_*rel*_.

**Figure 4:**
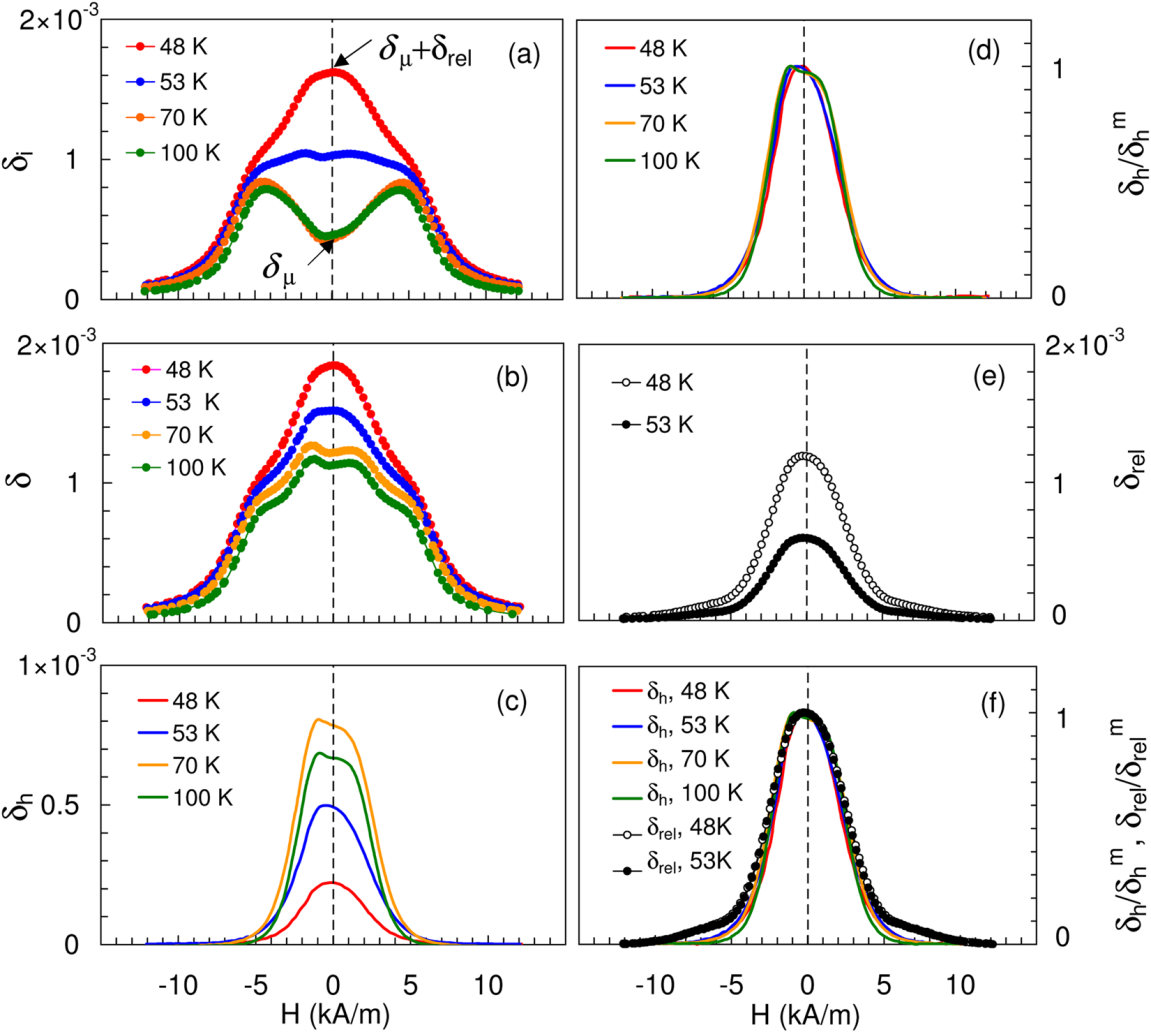
Effect of magnetic field on the total decrement, *δ*, its linear, *δ*_*i*_, relaxational, *δ*_*rel*_, and non-linear, *δ*_*h*_, components in Ni_80_Mn_20_ alloy at 70 and 100 K (well above *T*_*f*_) and at 53 and 48 K (near *T*_*f*_). (**a**) *δ*_*i*_ at strain amplitude of 10^−6^. (**b**) The total decrement *δ* at strain amplitude of 4 × 10^−5^. (**c**) *δ*_*h*_- the difference between the corresponding curves in (**b**) and (**a**). (**d**) Data of (**c**) normalized to the maximum value of the non-linear damping ${\delta }_{h}^{m}$ for each temperature. (**e**) *δ*_*rel*_ difference between *δ*_*i*_ registered at 48 and 53 K and the background *δ*_*i*_ at 70 K. (**f**) Data of (**e**) normalized to the maximum value of the relaxational damping component ${\delta }_{h}^{m}$ for each temperature with superimposed curves from (**d**).

The results of measurements of *δ* under cyclic *H* at fixed temperatures for Ni_80_Mn_20_ are shown in Fig. 4a,b. We selected four temperatures, two below *T*_*C*_ but above *T*_*f*_ (100 K and 70 K) and two near *T*_*f*_ (53 K and 48 K). At each temperature *δ*_*i*_(*H*) and *δ*(*H*) data were measured for low and high strain amplitudes and a linear field ramp with extrema *H*_*m*_ = ±12 kA/m. The damping at low strain amplitudes and at high amplitudes (including the sum of linear and non-linear components) approach zero as they saturate near *H*_*m*_ = 10 kA/m. The linear damping term, *δ*_*i*_, includes the micro- and macro-eddy terms and the relaxational damping *δ*_*rel*_:

1 $${\delta }_{i}(H)={\delta }_{\mu }(H)+{\delta }_{M}(H)+{\delta }_{rel}(H).$$

The difference between *δ*(*H*) registered at low, Fig. 4a, and high strain amplitude, Fig. 4b, is due to the contribution of the hysteretic MMD component, *δ*_*h*_. We now describe the effect of *H* on the various MMD components.

(i) Hysteretic damping *δ*_*h*_(*H*)

*δ*_*h*_(*H*) (Fig. 4c) is determined by subtracting the linear damping in Fig. 4a from the total damping (Fig. 4b). Each hysteretic MMD curve in Fig. 4(c) is then normalized by its highest value (Fig. 4d).

(ii) Micro- and Macro- eddy current damping *δ*_*μ*_ and *δ*_*M*_
  The side maxima of *δ*_*i*_ near *H* = 5 kA/m at 70 and 100 K and shoulders at 48 and 53 K in Fig. 4a,b, are independent of amplitude and occur for values of *H* below saturation. We identify them as due to the macro-eddy current component of damping *δ*_*M*_. Since *δ*_*M*_ = 0 for *M* = 0 *H* = 0, the points for *H* = 0 represent the microeddy MMD and a new relaxational damping, *δ*_*i*|*H = 0*_ = *δ*_*μ*_ + *δ*_*rel*_, Fig. 4a. The data on *δ*_*i*_(*H*) do not permit further distinctions between micro- and macro-eddy MMD contributions.
(iii) Relaxational damping *δ*_*rel*_(*H*)

The *δ*_*i*_(*H*) curves at 100 and 70 K do not include relaxational MMD components (Fig. 2b). They also do not differ significantly for different fields: *δ*_*i*_(100,*H*) ≈ *δ*_*i*_(70,*H*). Accordingly, we use the entire *δ*_*i*_(70,*H*) curve as a measure of the background for *δ*_*rel*_(*T*,*H*). The relaxational damping *δ*_*rel*_(53,*H*) and *δ*_*rel*_(48,*H*) is then determined as the difference *δ*_*rel*_(*T*,*H*) = *δ*_*i*_(*T*,*H*) − *δ*_*i*_(70,*H*) (Fig. 4e), using data from Fig. 4a. Each curve in Fig. 4e has been normalized by its highest value (Fig. 4f). Comparison with the normalized curves of Fig. 4d shows good agreement between *δ*_*h*_(*H*) and *δ*_*rel*_(*H*) over a large range of the *δ*_*h*_ and *δ*_*rel*_ suppression by *H*.

## Discussion

Typical MMD features occur at temperatures between *T*_*C*_ and *T*_*f*_, such as (i) an increased level of low amplitude linear damping *δ*_*i*_, related to micro-eddy current damping *δ*_*μ*_; (ii) the appearance of *δ*_*h*_ - the non-linear amplitude dependent hysteretic damping, (iii) existence of a peak in damping versus *H* that is independent of amplitude, which is the macro-eddy current peak, predicted by classical electromagnetic theory; macro-eddy damping disappears at *H* = 0 and *H* = *H*_*sat*_; (iv) emergence of the Δ*E* – effect, and (v) suppression of all three types of damping by magnetic field. This classic picture changes dramatically near RSG freezing, however. The key observation is the proportionality between *δ*_*rel*_(*H*) and *δ*_*h*_(*H*). Since *δ*_*h*_(*H*) is exclusively related to domain walls, the proportionality between *δ*_*rel*_(*H*) and *δ*_*h*_(*H*) suggests that *δ*_*rel*_ is also related to domain walls and, hence, the RSG transition is related to domain walls.

The behavior of Im (*Z*) in Fig. 2(a) and *δ*_*i*_(*T*) in Fig. 2(b) during RSG freezing agrees with the equivalent patterns of *χ*′(*T*) and *χ*″(*T*) in Ni_77_^57^Fe_1_Mn_22_^9^. In addition, the peak of *χ*″ is a linear function of the magnetic field^9^, just as *δ*_*rel*_ is linear in strain amplitude. Finally, all non-linear effects in *χ* freeze out during RSG freezing^9^, just as *δ*_*h*_ in the damping pattern. The only difference between our damping measurements and the susceptibility measurements in^9^ is that the *χ*″ peak during RSG freezing was found to be independent of *H* ^9^ while *δ*_*i*_ is completely suppressed by *H* in our work. A possible source of this disagreement is that the magnetic field employed in^9^ was below 400 A/m, which we find too low to show the field dependence of the relaxation, Fig. 4.

We propose, therefore, that the parallels between RSG-freezing of the susceptibility (*χ*′and *χ″*) and that of the anelasticity (ΔE-effect and damping) indicate the same physical origin, namely the freezing of domain wall motion. This idea implies that domain walls are the key structural elements responsible for RSG freezing instead of SG-like clusters^14,15^. This proposal is consistent with the results of detailed studies by Sato *et al*.^39^ on neutron depolarization and small angle scattering in Ni_77_Mn_23_. They identified the SG-like clusters, coexisting in the RSG state with FM order, as vortex-like structures. All the properties of these vortices are perfectly consistent with those usually attributable to DWs: (i) the size and the total volume of the vortices decrease with applied field; (ii) the vortex-like structures exist also in the FM state at *T* > *T*_*f*_, and are retained up to *T*_*C*_; (iii) the domain wall mobility decreases with lowering temperatures mainly due to freezing of spins in the vortices. The vortex-like structures always occur in complex arrays of domain walls as key ingredients of the RSG transition^40,41^.

The present interpretation agrees conceptually with the “domain glass” notion^21,22^, in the sense that RSG freezing reflects intrinsic domain wall properties: the “domain glass” concept considers “jamming” to be one of the mechanisms of glassiness in ferroic systems with developed domain wall structure and multiscale substructure. Observation of freezing out of linear microeddy current damping during RSG transition expands the concept of “domain glass” to freezing of infinitesimally small, atomic scale displacements of DWs. The domain wall immobilization is preceded by the transition from the non-thermally activated to the thermally activated mode of their motion. Such freezing, likely, should result from qualitative variations in domain wall structure at the atomic scale^42^.

The relaxation of DWs during RSG freezing, reported in the present work, is a new, fourth category of MMD, linear in strain amplitude as microeddy current MMD, but thermally activated in contrast to the non-thermally activated microeddy damping.

Among microscopic mechanisms for atomic-scale domain wall relaxations, is escape from a Peierls potential. The corresponding relaxation process implies thermally activated creation and motion of atomic-scale kinks^43–45^. However, this mechanism operates only for very narrow domain walls (high magneto-crystalline anisotropy), which is not applicable in our case.

The concept of a Peierls potential can be expanded to domain wall substructure, such as Bloch lines, able to control the DW mobility^46^. These linear structural entities can be susceptible to freezing similar to dislocations^47^ and to a transition from the non-thermally activated string-like behaviour to the thermally activated motion through creation and propagation of thermal kinks. In conclusion, an important consequence of the “domain glass” concept is that the glassy state is the consequence of intrinsic domain wall freezing and, therefore, may not necessarily require atomic structural disorder.

## Conclusions

Detailed acoustic studies of domain wall dynamics show that the re-entrant spin glass transition is related to freezing of domain walls. This conclusion implies a qualitatively new interpretation of re-entrant spin glass transitions. The main features of the freezing process are:

- freezing is marked by a domain-wall related, relaxational linear magneto-mechanical damping peak, which is a new category of magnetomechanical damping;
- cooling through the freezing temperature is seen as the transition from the non thermally activated mode of domain wall motion to the thermally activated mode followed by a complete immobilization of domain walls on both atomic and mesoscopic scales;
- domain wall freezing implies variation of their structure on an atomic scale, resulting in complex spin arrangement and their immobilization. Specific mechanisms of domain wall freezing might include partitioning of domain walls into many low-symmetry variants or freezing of such elements of their internal substructure as the Bloch line in a Peierls-lattice potential.

## Materials and Methods

Ingots of Ni-Mn alloys were produced by induction casting from 99.99 wt% purity components and homogenized for 24 hrs at 1170 K, followed by water quenching. Samples with dimensions approximately 1.5 × 1.5 × 24 mm were cut by spark erosion.

A 10 g ingot of the Au_86_Fe_14_ alloy was produced from 99.99 wt% purity ingredients. Fe pellets of appropriate mass were wrapped in Au foil and the cigar thus formed was sealed in an evacuated quartz tube, melted and water quenched. The ingot was re-melted 3 times, also in evacuated quartz tubes, and finally annealed at 1170 K for 24 hrs. The tube with the annealed ingot was taken rapidly from the furnace and the ingot was quenched in water immediately after breaking the quartz tube. Samples for ultrasonic experiments measured 1.5 × 1.5 × 12 mm. Compositions of all alloys were checked by EDS.

Acoustic measurements were performed by three component piezoelectric composite oscillator technique^48^ at a frequency near 90 kHz. The experimental arrangement is described elsewhere^49^. It allows the determination of the logarithmic decrement, *δ*, and the resonant frequency, *f*, of the fundamental mode of the longitudinal oscillations of the sample. The effective Young’s modulus, *E*, was calculated from the resonant frequency, the density *ρ* and the length *l* of the sample, as *E* = 4*ρl* ^2^ *f* ^2^.

In the first type of acoustic experiments, temperature spectra of the Young’s modulus, strain amplitude-independent *δ*_*i*_ and strain amplitude dependent internal friction *δ*_*h*_ between 15 and 300 K were registered simultaneously in the same temperature scan. Two values of the oscillatory strain amplitude *ε*_0_ (low, *ε*_0_ = 10^−6^, and high, *ε*_0_ = (2–4) × 10^−5^) were alternately stabilized for each temperature^50^. The strain amplitudes correspond to amplitude-independent and amplitude-dependent ranges of damping, respectively. This way, the linear damping versus temperature, *δ*_*i*_(*T*), was directly registered for *ε*_0_ = 10^−6^. The damping measured at high strain amplitude contained both linear *δ*_*i*_ and non-linear *δ*_*h*_ components: *δ*(*T*) = *δ*_*i*_(*T*) + *δ*_*h*_(*T*). The non-linear damping temperature spectra were obtained as the difference between the spectra at high and low strain amplitudes: *δ*_*h*_(*T*) = *δ*(*T*) − *δ*_*i*_(*T*). Damping and Young’s modulus temperature spectra were recorded under different fixed polarizing magnetic fields *H*, including *H* = 0.

In a second experimental protocol, the dependences of ultrasonic absorption on *ε*_0_ were measured at different fixed temperatures. Finally, *δ* and *E* were measured for fixed temperatures under cyclic polarizing field at constant *ε*_0_ in the ranges of linear and non-linear an-elastic behavior.

Temperature, strain amplitude, and field spectra were measured with an Oxford closed-loop cryostat equipped with a heater free of magnetic fields and modified for acoustic studies with a temperature rate of 2 K/min. The oscillator was placed inside a closed copper chamber and the temperature of the sample was determined by means of an additional Lakeshore Cernox sensor placed in close vicinity (within 1–2 mm) to the sample. The samples were demagnetized thermally by cooling below the Curie temperature under zero field. The measurements of *δ*(*ε*_0_) dependences at different temperatures were done consecutively in one heating scan, that is, without demagnetization between each measurement. A polarizing magnetic field was created by a solenoid 40 cm long, 5 cm in diameter, providing homogeneity of the field in the working space of a sample of better than 1%. A triangularly shaped wave was used to create a periodic magnetic field, parallel to the sample’s long axis, up to ±12 kA/m. The frequency of the applied field, 5 × 10^−4^ Hz, was sufficiently low to record 200 internal friction and frequency data points versus field in each cycle.

Measurements of electric impedance, *Z* = Re(*Z*) + *i* Im(*Z*), for the same samples as used for ultrasonic studies, served to determine characteristic temperatures of magnetic transitions from the behavior of the imaginary part of the impedance, which, in such experiments and as a first approximation, can be considered proportional to the magnetic permeability *µ*: Im(*Z*) ∝ *μ* ^51^. A standard four-terminal method with an excitation frequency 217 Hz and 686 Hz was used.
